# A Latent Variable Mixed-Effects Location Scale Model with an Application to Daily Diary Data

**DOI:** 10.1007/s11336-022-09864-8

**Published:** 2022-05-03

**Authors:** Shelley A. Blozis

**Affiliations:** Department of Psychology, University of California, One Shields Avenue, Davis , CA 95616 USA

**Keywords:** second-order latent variable model, positive affect, intraindividual variation, latent state trait theory

## Abstract

A mixed-effects location scale model allows researchers to study within- and between-person variation in repeated measures. Key components of the model include separate variance models to study predictors of the within-person variance, as well as predictors of the between-person variance of a random effect, such as a random intercept. In this paper, a latent variable mixed-effects location scale model is developed that combines a longitudinal common factor model and a mixed-effects location scale model to characterize within- and between-person variation in a common factor. The model is illustrated using daily reports of positive affect and daily stressors for a large sample of adult women.

**Supplementary Information:**

The online version contains supplementary material available at 10.1007/s11336-022-09864-8.

Psychological investigations that rely on repeated-measures study designs typically aim to study within- and between-person variation in a response over repeated occasions. Popular models for these kinds of problems are latent curve and mixed-effects models (Bollen and Curran 2006; Singer and Willett 2003; Skrondal and Rabe-Hesketh 2004). Often these models are applied to describe the typical longitudinal trajectory of a population and individual differences in the features that describe the trajectories (e.g., Ricker et al. 2018). Other investigations additionally include study of the within-person variation of the repeated measures (e.g., Kim and Cicchetti 2009). For time-intensive data collections over relatively short periods of time, the study of within-individual variation may be the primary focus (e.g., Allaire and Marsiske 2005). Many applications of latent curve and mixed-effects models have emphasized inference from the subject-specific model and the random effects that distinguish the individual trajectories, often while assuming that the errors in the occasion-level model have equal variance, not only across measurement occasions but across individuals. Otherwise, a number of options, largely restricted to data that are equally spaced with regard to time, in specifying the residual covariance structure may be considered to better represent variation (and possibly covariation) of scores within individuals, even if only to improve inference about the fixed effects of the model and individual differences in the coefficients of the subject-specific model. In some types of investigations that rely on repeated measures data, heterogeneity of the occasion-level residual variance may be a particularly interesting feature of the response, potentially offering further insight into individual differences in repeated measures. Depending on a study’s goals, examination of both within- and between-person variation in repeated measures data can be important in fully understanding and characterizing psychological variables (Molenaar 1985; Nesselroade 2004).

A mixed-effects location scale model offers a way to explicitly model within- and between-person variation in repeated measures data and possesses key features that distinguish it from typical formulations of a latent curve or mixed-effects model. An extension of a standard mixed-effects model, a mixed-effects location scale model includes a model for the variance of a random coefficient relating to the subject-specific model and a model for the variance of the residual relating to the occasion-level model (Hedeker et al. 2008). Under this model, the variance of a random coefficient and the variance of the occasion-level residual can depend on covariates. Notably, the model for the occasion-level residual variance additionally includes a random subject effect to allow for between-person differences in the occasion-level residual variance. Motivations for using a mixed-effects location scale model may stem from a need to explicitly model heterogeneity of variance within, as well as between, individuals.

Similar to applications of latent curve and mixed-effects models that assume the response data are free of measurement error, a mixed-effects location scale model does not account for measurement error in response data. Given scores measured without error, the variance of the occasion-level residual will reflect true variation of scores within subject. If the scores include measurement error, however, the residuals will reflect a combination of within-subject variation from the fitted model and measurement error. This can pose a significant problem in evaluating how well a model fits the observed data, but importantly for investigations that include study of the within-subject variation, added measurement error can naturally inflate the magnitude of the within-subject variation (e.g., Goldstein et al. 2018; Hedeker et al. 2012; Rast et al. 2012). Measurement error has been addressed in latent curve and mixed-effects models by relying on a common factor model to represent the manifest variables, with a latent growth model applied to the latent variables (Blozis 2007; Harring 2009; McArdle 1988; McNeish and Dumas 2017; Sayer and Cumsille 2001). Given the benefits of a latent variable model, the mixed-effects location scale model can benefit from a latent variable approach. To capitalize on the flexibility of a mixed-effects location scale model to allow for within- and between-person heterogeneity of variance and on a latent variable model to address measurement error, this paper uses a latent variable model to expand a mixed-effects location scale model for repeated measures of a latent variable.

## Repeated Measures from Daily Diary Studies

**Fig. 1:**
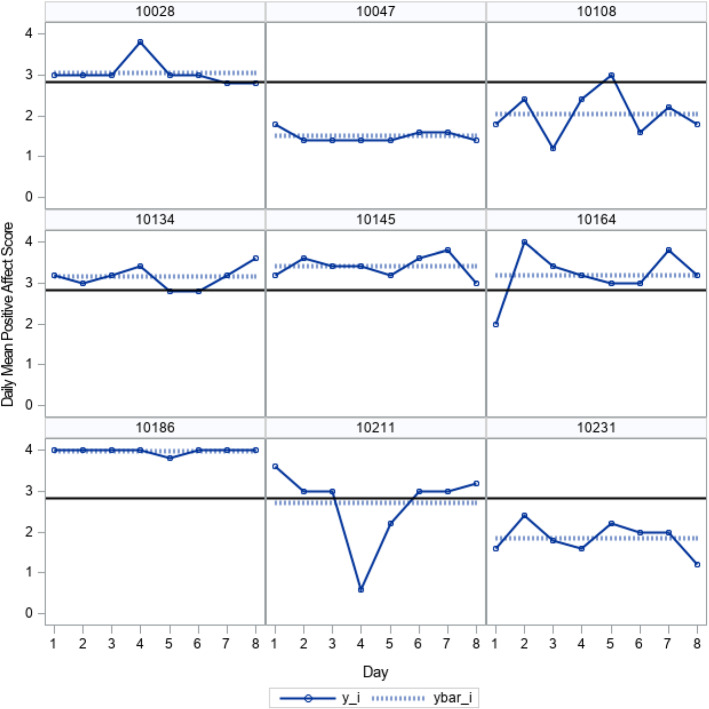
Daily positive affect scores $(y_i)$ and daily positive affect scores averaged across days $(ybar_i)$ for 9 study participants, plotted with the mean score (2.8) across days and all participants.

Data from daily diary studies often display fluctuations in scores within individuals. An example is a set of daily positive affect scores for 9 individuals shown in Fig. 1 that come from the Midlife in the United States (MIDUS 2): Daily Stress Project (Ryff and Almeida 2004-2009). The data are part of a larger longitudinal study that began in 1995 (MIDUS 1) and included a nationally representative random-digit-dial sample of noninstitutionalized, English-speaking adults residing in the contiguous USA. Participants of MIDUS 1 were invited to participate in a second longitudinal wave (MIDUS 2) about 10 years later, along with a newly added adult cohort. From MIDUS 2, a subset of participants were selected at random to participate in the Daily Stress Project. Participants were interviewed by telephone to obtain daily self-reports for 8 consecutive days. Multiple scales were administered daily to measure different psychological constructs. Here, responses for 435 women with complete data on 5 survey items designed to reflect daily positive affect are considered. For instance, the first item asked "Did you feel in good spirits?" Items were measured using a 5-point ordinal response scale: 0=none of the time to 4=all of the time. In Fig. 1 are daily scores for 9 women, with scores computed as the daily mean of the 5 scale items. Calculating an average or summed score is a common way to handle responses obtained for a set of items designed to measure a common construct (e.g., Hedeker et al. 2012; Rast et al. 2012). Although a measurement model for these items is provided later in Section 4, it is useful to first consider the usual approach to handling responses to multiple items. As shown in Fig. 1, the solid horizontal line that is common to all panels of the display is the mean score of 2.8 calculated across days and women. The dashed horizontal line unique to each plot is the individual’s response averaged across days. From the display, it can be seen that within individuals, scores fluctuate from day to day relative to the individual’s mean, and the extent of the daily variation within person varies between people. The mean responses of individuals differ from the overall mean and from each other. It is also interesting to note that for some individuals, scores reach the maximum possible score of 4 (Case 10186) while also displaying vary little variation across days. Although standard mixed-effects model might be effective in characterizing the differences between individuals with regard to their mean affect levels, the model would not address the between-subject heterogeneity of the intraindividual variation. Indeed, it is the between-subject differences in the within-subject variation that is a notable feature of the affect scores. Thus, a mixed-effects location scale model that permits the within-subject variance to vary by subject would seem to be quite suitable in characterizing the scores. This, in particular, would help to address possible ceiling effects in measurement (Hedeker et al. 2012).

Common to many studies of a psychological variable is an implicit assumption that the variable is composed of trait- and state-level components (Nesselroade 1987). For example, latent state trait theory suggests that a psychological measure is comprised of three independent sources: a latent trait, a latent state residual and measurement error (Steyer et al. 1992). The latent trait component reflects the part of the score that is not specific to the measurement occasion, but rather, is unique to the person. Conversely, the latent state residual component is specific to the measurement occasion and the individual and, as such, is expected to fluctuate about the trait-level component according to the measurement occasions. Importantly, both the latent trait and state components are assumed to be free of measurement error. To permit study of the two latent components of a psychological variable, a second-order model can be applied to account for measurement error in the observed measures (Steyer et al. 1992). An interesting application of a mixed-effects location scale model is to problems such as the study of daily positive affect (Hedeker et al. 2012; Rast et al. 2012). The model provides a framework for separating the trait and state components of a measured variable (albeit assumed to be measured without error) while allowing the state residual to be truly unique to the individual by inclusion of a random subject effect in a model for the occasion-level residual variance (i.e., state residual variance). However, the standard formulation of the model does not address measurement error. To address the issue of measurement error in applications of latent state trait theory, a common factor model is applied to repeated measures data. The variance of a general common factor is assumed to be due to the latent trait factor and the variance of the occasion-specific factor is assumed to be due to the latent state residual factor. Thus, a potentially useful model is a latent variable mixed-effects location scale model that would permit partitioning of the observed item variance into the distinct components, and additionally, provide a truly person- and occasion-specific measure of the state residual variance.

## The Mixed-Effects Location Scale Model

A two-level linear mixed-effects location scale model is described here (for a 3-level model, see Lin et al. 2018; for a generalized linear version of the model, see Hedeker et al. 2016). Let $y_{t i}$ be an observed response at time $t =1 ,\ldots ,n_{i}$, for individual *i*, $i =1 ,\ldots ,N$, where $n_{i}$ denotes that the number of observations can vary by the individual. Missing data are assumed to be missing at random. A linear mixed-effects location scale model for $y_{t i}$ can be given as (cf: Hedeker et al. 2008):

1 $$\begin{aligned} y_{t i} =\mathbf {x}_{t i}^{ \prime } (\mathbf {\beta }_{x} +\mathbf {\upsilon }_{i}) +\mathbf {w}_{i}^{ \prime } \mathbf {\beta }_{w} +\varepsilon _{t i} , \end{aligned}$$

where $\mathbf {x}_{t i}$ is a *p* x 1 vector of covariates that vary according to the measurement occasion *t* and individual *i* and would usually include a "1" for the intercept of model, and $\mathbf {w}_{i}$ is a *q* x 1 vector of covariates that vary according to the individual *i*. The coefficient $\mathbf {\beta }_{x}$ is a set of *p* fixed regression coefficients linking $\mathbf {x}_{t i}$ to $y_{t i}$, and $\mathbf {\upsilon }_{i}$ is a set of $p^{ *} \le p$ random subject effects that correspond to one or more of the fixed effects in $\mathbf {\beta }_{x}$. The coefficient $\mathbf {\beta }_{w}$ is a set of *q* fixed regression coefficients linking $\mathbf {w}_{i}$ to $y_{t i}$. The random effect $\mathbf {\upsilon }_{i}$ is assumed to be independent and normally distributed across individuals as

2 $$\begin{aligned} \mathbf {\upsilon }_{i} \sim N \left( \mathbf {0} ,\mathbf {\Phi }_{\upsilon } \left( \mathbf {w}_{i} ,\mathbf {\alpha } ,\mathbf {\rho }_{\upsilon }\right) \right) , \end{aligned}$$

where the between-subject covariance matrix $\mathbf {\Phi }_{\upsilon }$ may be a function of between-subject covariates ($\mathbf {w}_{i}$) and corresponding coefficient vector $\mathbf {\alpha }$, with $\mathbf {\alpha } =(\alpha _{0} ,\ldots ,\alpha _{q}$) denoting the set of coefficients that link the covariates to elements of the matrix $\mathbf {\Phi }_{\upsilon }$. The set of coefficients $\mathbf {\rho }_{\upsilon }$ $=\left( \rho _{1} ,\ldots \right)$ are the correlations between the random effects conditional on $\mathbf {w}_{i}$. For instance, a common application of a mixed-effects location scale model is one in which only the intercept is random (e.g., Hedeker et al. 2008), and so $\mathbf {\Phi }_{\upsilon } =\phi _{\upsilon _{i}}^{2}$ with the variance of the random intercept modeled as

3 $$\begin{aligned} \phi _{\upsilon _{i}}^{2} =\exp \left\{ \alpha _{0} +\alpha _{1} w_{1 i} +\ldots +\alpha _{q} w_{q i}\right\} , \end{aligned}$$

where $\alpha _{0}$, when exponentiated, is the variance of $\upsilon _{i}$ conditional on $\mathbf {w}_{i}$. The remaining coefficients are the between-subject covariate effects on the between-subject variance. A positive effect of a coefficient indicates that a unit increase in a covariate corresponds to an increase in the variance of the random effect; a negative effect indicates that a unit increase in a covariate corresponds to a decrease in the variance. As will be shown later using an empirical example, allowing a between-subject variance to be a function of covariates makes it possible address between-subject heterogeneity of variance in a random effect.

At the occasion level, the residual $\varepsilon _{t i}$ in (1) is assumed to be independent and normally distributed as:

4 $$\begin{aligned} \varepsilon _{t i} \sim N \left( 0 ,\sigma _{\varepsilon }^{2} \left( \mathbf {x}_{t i} ,\mathbf {w}_{i}, \mathbf {\tau }\text {,}a_{i}\right) \right) , \end{aligned}$$

where the variance $\sigma _{\varepsilon }^{2}$ may be a function of $\mathbf {x}_{t i}$ (that would usually include a "1" for the intercept) and $\mathbf {w}_{i}$, with corresponding coefficient vector $\mathbf {\tau } =(\mathbf {\tau }_{x} ,\mathbf {\tau }_{w})$, where $\mathbf {\tau }_{x} =(\mathbf {\tau }_{x 1} ,\ldots ,\mathbf {\tau }_{x p})$ and $\mathbf {\tau }_{w} =(\tau _{w 1} ,\ldots ,\tau _{w q}$) denoting sets of coefficients that link the within-subject and between-subject covariates, respectively, to the within-subject variance $\sigma _{\varepsilon }^{2}$. In (4), the coefficient $a_{i}$ denotes a between-subject random effect. Specifically, the variance of $\varepsilon _{t i}$ is modeled as

5 $$\begin{aligned} \sigma _{\varepsilon }^{2} =\exp \left\{ \tau _{0} +\tau _{x 1} +\ldots +\tau _{x p} +\ldots +\tau _{w 1} +\ldots +\tau _{w q} +a_{i}\right\} , \end{aligned}$$

where $\tau _{0}$, when exponentiated, is the residual variance conditional on the covariates and for a subject for whom $a_{i} =0$. Similar to the interpretation of the covariate effects in (2) that serve to account for between-subject heterogeneity of variance in a random effect, a positive value for the effect of a covariate in the model for the within-subject variance indicates that a unit increase in a covariate corresponds to an increase in the residual variance, and a negative value indicates that a unit increase in a covariate corresponds to a decrease in the variance. As will be shown later using an empirical example, allowing the within-subject variance to be a function of covariates makes it possible address heterogeneity of the individual and occasion-specific residual variance.

The model for the variance of the occasion-specific residual $\varepsilon _{t i}$ in (4) includes $a_{i}$ to address heterogeneity of variance due to unobserved effects. That is, the variance of $\varepsilon _{t i}$ may differ between individuals, even after conditioning on covariates, and so including the random effect $a_{i}$ allows for heterogeneity that may remain. This may be important, for instance, in problems for which there are between-subject differences in the variability in scores across occasions, such as if a ceiling effect in the measurement of the response is present and this results in reduced variation in scores for individuals whose average scores are at the extremes of the response scale. The random effect is assumed to be independent and normally distributed as

6 $$\begin{aligned} a_{i} \sim N \left( 0 ,\phi _{a}^{2}\right) . \end{aligned}$$

At the subject level of the model, the random effect $\mathbf {\upsilon }_{i}$ in (1) and the random effect $a_{i}$ in (4) may covary. To allow this, the random effects are stacked, with the set defined here as $\mathbf {c}_{i} \left( r\text {x}1\right) =\left( \mathbf {\upsilon }_{i}^{ \prime } ,a_{i}\right) ^{ \prime }$ to include all $r =p^{ *} +1$ random effects. It is assumed that the joint distribution of $\mathbf {c}_{i}$ is

7 $$\begin{aligned} \mathbf {c}_{i} \sim N \left( \mathbf {0} ,\mathbf {\Phi }_{c}\right) , \end{aligned}$$

where the covariance matrix is

8 $$\begin{aligned} \mathbf {\Phi }_{c} =\left( \begin{array}{cc}\mathbf {\Phi }_{\upsilon } \left( \mathbf {w}_{i} ,\mathbf {\alpha } ,\mathbf {\rho }_{\upsilon }\right) &{} \mathbf {\phi }_{\upsilon a}^{{}} \\ \mathbf {\phi }_{\upsilon a}^{ \prime } &{} \phi _{a}^{2}\end{array}\right) . \end{aligned}$$

It follows that the probability distribution of $\mathbf {c}_{i}$ is

9 $$\begin{aligned} h_{c} \left( \mathbf {c}_{i}\right) =2 \pi ^{r/2} \left| \mathbf {\Phi }_{c}\right| ^{ -1/2} \exp \left( \left( \mathbf {c}_{i} -\mathbf {\mu }_{c}\right) ^{ \prime } \mathbf {\Phi }_{c}^{ -1} \left( \mathbf {c}_{i} -\mathbf {\mu }_{c}\right) \right) . \end{aligned}$$

Letting $\mathbf {y}_{i} =\left( y_{1 i} ,\ldots ,y_{n_{i}}\right) ^{ \prime }$and conditional on $\mathbf {c}_{i}$, the distribution of $\mathbf {y}_{i}$ is

10 $$\begin{aligned} \mathbf {y}_{i}\vert \mathbf {c}_{i} \sim N \left( \mathbf {\mu }_{y\vert c} ,\mathbf {\Sigma }_{y\vert c}\right) , \end{aligned}$$

where

11 $$\begin{aligned} \mathbf {\mu }_{y\vert c} =\mathbf {\Gamma }_{i} \left( \mathbf {x}_{t i} ,\mathbf {w}_{i}\right) \mathbf {\beta } \end{aligned}$$

and

12 $$\begin{aligned} \mathbf {\Sigma }_{y\vert c} =\mathbf {\Gamma }_{i} \left( \mathbf {x}_{t i} ,\mathbf {w}_{i}\right) \mathbf {\Phi }_{\upsilon } \left( \mathbf {w}_{i} ,\mathbf {\alpha } ,\mathbf {\rho }_{\upsilon }\right) \mathbf {\Gamma }_{i} \left( \mathbf {x}_{t i} ,\mathbf {w}_{i}\right) ^{ \prime } +\mathbf {\Theta }_{\varepsilon } \left( \mathbf {x}_{t i} ,\mathbf {w}_{i} , \mathbf {\tau },\,a_{i}\right) , \end{aligned}$$

where $\mathbf {\Gamma }_{i} \left( \mathbf {x}_{t i} ,\mathbf {w}_{i}\right)$ is a design matrix containing the within- and between-subject covariates, $\mathbf {\beta } =\left( \mathbf {\beta }_{x}^{ \prime } ,\mathbf {\beta }_{w}^{ \prime }\right) ^{ \prime }$ is a stacked set of fixed coefficients linking the covariates to $\mathbf {y}_{i}$, and assuming independence of the occasion-specific residuals between occasions and subjects, $\mathbf {\Theta }_{\varepsilon } \left( \mathbf {x}_{t i} ,\mathbf {w}_{i} ,\mathbf {\tau }\text {,}a_{i}\right) =\mathbf {I}_{n_{i}} \sigma _{t i}^{2} \left( \mathbf {x}_{t i} ,\mathbf {w}_{i} ,\mathbf {\tau }\text {,}a_{i}\right)$, with $\mathbf {I}_{n_{i}}$ being an identity matrix of order $n_{i}$. Then, the conditional probability density function can be defined as

13 $$\begin{aligned} h_{y\vert c} \left( \mathbf {y}_{i}\vert \mathbf {c}_{i}\right) =2 \pi ^{ -p/2} \left| \mathbf {\Sigma }_{y\vert c}\right| ^{ -1/2} \exp \left( \left( \mathbf {y}_{i} -\mathbf {\mu }_{y\vert c}\right) ^{ \prime } \mathbf {\Sigma }_{y\vert c}^{ -1} \left( \mathbf {y}_{i} -\mathbf {\mu }_{y\vert c}\right) \right) , \end{aligned}$$

and the marginal distribution of $\mathbf {y}_{i}$ is then

14 $$\begin{aligned} g \left( \mathbf {y}_{i}\right) ={\displaystyle \int _{\mathbf {c}_{i}}}h_{y} \left( \mathbf {y}_{i}\vert \mathbf {c}_{i}\right) h_{c} \left( \mathbf {c}_{i}\right) d \mathbf {c}_{i} . \end{aligned}$$

The parameters of the model are $\mathbf {\gamma } =\left( \mathbf {\beta } ,\mathbf {\alpha } ,\mathbf {\tau } ,\mathbf {\phi }_{\upsilon } ,\mathbf {\phi }_{\upsilon a} ,\phi _{a}^{2}\right)$. Given a random sample of observations, $\mathbf {y}_{1} ,\ldots ,\mathbf {y}_{N}$, the marginal log-likelihood of $\mathbf {\gamma }$ is

15 $$\begin{aligned} \text {ln}L \left( \mathbf {\gamma }\right) ={\displaystyle \sum _{i =1}^{N}}\ln g \left( \mathbf {y}_{i}\right) . \end{aligned}$$

The model for $\mathbf {y}_{i}$ is nonlinear in the scale random effect, $a_{i}$, and consequently, the integral in (14) does not have a closed-form expression. Estimation of the integral generally would rely on an approximation to the integral (Pinheiro and Bates 1995), such as by implementing SAS$^{1}$ PROC NLMIXED that permits random effects to be incorporated into the within-subject covariance matrix in (4) (see Hedeker et al. 2012).

### Example: Manifest Measures of Daily Mean Positive Affect

The daily mean positive affect scores described earlier for the 435 women are analyzed using a mixed-effects location scale model (Hedeker et al. 2012; Rast et al. 2012). Within individuals, daily measures of positive affect may not be expected to change systematically with the passage of days, but rather, vary across days. Additionally, scores averaged across days within individuals would be expected to naturally vary between individuals. Accordingly, the manifest measures of mean positive affect are assumed to follow a random intercept model:

$$\begin{aligned} y_{t i} =\beta _{0 i} +\varepsilon _{t i}\text {,} \end{aligned}$$

where, for participant *i*, $y_{t i}$ is the observed mean positive affect score on day *t*, $\beta _{0 i} =\beta _{0} +\upsilon _{i}$ is the expected response across days, and $\varepsilon _{t i}$ is the discrepancy between the observed and fitted responses on day *t*. The random intercept $\beta _{0 i}$ represents the trait component that varies by individual, and the residual $\varepsilon _{t i}$ represents the state component that varies by both individual and occasion. The random intercept is assumed to be normally distributed: $\beta _{0 i} \sim N \left( \beta _{0} ,\phi _{\upsilon }^{2}\right)$, where

$$\begin{aligned}\phi _{\upsilon }^{2} =\exp \left\{ \alpha _{0}\right\} \text {.} \end{aligned}$$

This expression of the variance of the random intercept becomes useful later when covariates are added to the model to address between-subject heterogeneity in the random intercept variance. The residual is assumed to be normally distributed: $\varepsilon _{t i} \sim N \left( 0 ,\mathbf {I} \sigma _{\varepsilon }^{2}\right)$, where

$$\begin{aligned}\sigma _{\varepsilon }^{2} =\exp \left\{ \tau _{0} +a_{i}\right\} \text {,} \end{aligned}$$

and $a_{i}$ is a random subject effect that is assumed to be normally distributed: $a_{i} \sim N \left( 0 ,\phi _{a}^{2}\right) \text {.}$ At the subject level, the random intercept and the random effect of the within-subject variance may covary: $c o v \left( \upsilon _{i} ,a_{i}\right) =\phi _{\upsilon a}^{{}}$. The model has five parameters: $\mathbf {\gamma } =\left( \beta _{0} ,\alpha _{0} ,\tau _{0} ,\phi _{\upsilon }^{2} ,\phi _{\upsilon a}^{{}}\right) \text {.}$

To assess the need to include the random effect in the model for $\sigma _{\varepsilon }^{2}$, estimates were obtained for a reduced form of the model, henceforth called Model A$_{1}$, that assumed homogeneity of the residual variance, and so $\phi _{\upsilon }^{2} =0$ (1st set of estimates in Table 1 corresponding to Model A$_{1}$). Thus, Model A$_{1}$ is a random intercept model with a fixed intercept $\beta _{0}$ and includes parameter $\alpha _{0}$ of the variance model for the random intercept ($\phi _{\upsilon }^{2} =\exp \left\{ \alpha _{0}\right\}$), and parameter $\tau _{0}$ of the variance model for the residual ($\sigma _{\varepsilon }^{2} =\exp \left\{ \tau _{0}\right\}$). Model A$_{1}$ and the mixed-effects location scale model, henceforth called Model A$_{2}$, were fit using SAS PROC NLMIXED version 9.4 using nonadaptive Gaussian quadrature with 7 quadrature points. Increasing the number of quadrature points beyond 7 or using a Laplacian approximation (Vonesh 1996) did not result in a converged solution for Model A$_{2}$. Maximum likelihood (ML) estimates of Model A$_{2}$ are given in Table 1 (second set of estimates). Starting values for the estimation of these models involved fitting a fixed-effects model first, followed by adding a random intercept (for Model A$_{1}$), then a random effect in the within-subject variance model and the covariance between it and the random intercept (for Model A$_{2}$). ML estimation carried out using SAS PROC NLMIXED with a Laplacian approximation). Model A$_{2}$ that assumed $\phi _{\upsilon }^{2} >0$ yields an improvement in model fit in terms of the AIC, from 6, 087 (Model A$_{1}$) down to 5, 547 (Model A$_{2}$) suggesting between-subject heterogeneity of the within-person variance. A likelihood ratio test (LRT) of the goodness-of-fit between the two models using 2 df is $\chi _{2}^{2} =6,081 -5,537 =544\text {,}$ $p <.001\text {,}$ supporting the need to include the random effect in the occasion-level residual variance model. From Model A$_{2}$, the estimated common variance of the residual is $\exp ( -1.84) =0.16$, and the estimated standard deviation of the random effect in the model for $\sigma _{\varepsilon }^{2}$ is 1.16. These results suggest between-subject differences in the extent to which daily positive affect scores vary from day to day. The estimated correlation between the random intercept and the random subject effect for the within-subject variance is $r = -.36$, suggesting a tendency for those with a relatively low day-to-day variation in positive affect scores to also have a relatively high positive affect score averaged across days. Thus, either those with relatively high levels of positive affect across days are also relatively stable in their affect, or the result is an indication of a ceiling effect in the measurement. Finally, from the second of the two models, the estimated mean positive affect score across days and participants is $\hat{\beta }_{0} =3.00$ $\left( s e =0.006\right)$, indicating a rather high average across days and participants.

**Table 1:** ML Estimates of a mixed-effects location scale model of manifest measures of daily mean positive affect ($n=435$).

	Model A$_{1}^{a}$	Model A$_{2}^{b}$	Model A$_{3}^{b}$
*Mean structure*			
$\beta _{0},$ intercept	2.81(0.032)	$3.00\left( 0.006\right)$	$3.05\left( 0.012\right)$
$\beta _{1}$, DS$_{ti}^{*}$			$-0.075\left( 0.013\right)$
$\beta _{2}$, DMS$_{i}$			$-0.36\left( 0.030\right)$
*Covariance structure*			
*Between-subject*			
$\alpha _{0}$, intercept	$-0.90(0.073)$	$-0.65(0.039)$	$-1.81\left( 0.026\right)$
$\alpha _{1}$, DMS$_{i}$			$0.36\left( 0.070\right)$
$\phi _{a}$		1.16(0.056)	$1.11\left( 0.053\right)$
$\rho _{\upsilon a}$		$-0.36(0.061)$	$-0.34\left( 0.042\right)$
*Within-subject*			
$\tau _{0}$, intercept	$-1.42(0.026)$	$-1.84(0.065)$	$-2.29\left( 0.10\right)$
$\tau _{1}$, DS$_{ti}^{*}$			$0.30\left( 0.049\right)$
$\tau _{2}$, DMS$_{i}$			$0.78\left( 0.15\right)$
Deviance	6081	5537	5245
AIC	6087	5547	5265

Notes:$\ ^{a}$ML estimation carried out using SAS PROC NLMIXED with a Laplacian approximation to the integral. $^{b}$ML estimation carried out using SAS PROC NLMIXED with nonadaptive Gaussian quadrature with 7 quadrature points

#### Daily Stressors

Extensive research has demonstrated a clear relationship between negative affect and daily stress, but positive reactivity to daily stress is not as well understood. Several studies report mixed findings in terms of the direction of the relationship, suggesting that the relationship is complex and highly individualistic (Schilling and Diehl 2014). Here, the mixed-effects location scale model is extended to include the number of daily stressors reported for each interview day, denoted by *daily stressors*$_{t i}$. Scores were based on a sum of responses to 7 questions scored as 1=yes and 0=no; the first in the set of questions, for instance, was “Did you have an argument or disagreement with anyone since (this time/we spoke) yesterday?” The possible minimum and maximum scores were 0 and 7, respectively. Given repeated measures of the number of stressors, both the within- and between-subject effects of daily stressors on daily mean positive affect scores were studied. To do this, the variable was centered about the individual’s mean number of stressors across days: *DS*$_{t i}^{ *}$= *daily stressors*$_{t i}- \textit{DS}_{ .i}$), where *DS*$_{t i}^{ *}$ denotes an individual’s daily stress count centered about their mean count across days, with the latter denoted by *DS*$_{ .i}$. Daily stressor counts for the sample ranged from 0 to 5; individual-level mean counts ranged from 0 to 2.38, with an overall mean of 0.53 and standard deviation of 0.42. The person-mean centered and mean values of the daily stressor measures were included in the model to predict the observed daily mean positive affect score:

$$\begin{aligned}y_{t i} =\beta _{0 i} +\beta _{1} \text {\textit{DS}}_{t i}^{ *} +\beta _{2} \text {\textit{DS}}_{ .i} +\varepsilon _{t i}\text {.} \end{aligned}$$

To evaluate the effect of daily stressors on the variation of positive affect scores within and across days, after adjusting for the within- and between-person effects of daily stressors, both measures of daily stressors were included in the model of the occasion-level residual variance:

$$\begin{aligned}\sigma _{\varepsilon }^{2} =\exp \left\{ \tau _{0} +\tau _{1} \text {\textit{DS}}_{t i}^{ *} +\tau _{2} \text {\textit{DS}}_{ .i} +a_{i}\right\} \text {.} \end{aligned}$$

Thus, this model was used to evaluate whether higher daily counts of stressors or an overall high level of stressors across days was related to the daily variation in mean positive affect scores across days. The variance of the random intercept was also evaluated for between-subject heterogeneity by including the mean daily stressor count in the model of the random intercept variance:

$$\begin{aligned} \phi _{\upsilon }^{2} =\exp \left\{ \alpha _{0} +\alpha _{1} \text {\textit{DS}}_{ .i}\right\} \text {.} \end{aligned}$$

Starting values for the effects of covariates were set to 0, and the remaining parameters were set equal to the values obtained from fitting the unconditional model described in the previous section. The model was fit using SAS PROC NLMIXED version 9.4 using nonadaptive Gaussian quadrature with 7 quadrature points. Estimates of the conditional model, referred to as Model A$_{3}$, are in the last column of Table 1. The mean positive affect score for those with no daily stressors is estimated to be 3.05 ($s e =.012$), a value that is slightly greater than that obtained under Model A$_{2}$. The estimates further suggest an overall negative within-person effect ($\hat{\beta }_{1} = -0.075$, $s e =0.012$), as well as a negative between-person effect ($\hat{\beta }_{2} = -0.36$, $s e =0.030$), of the number of daily stressors on daily mean positive affect, with the magnitude of the between-person effect being nearly five times the size of the within-person effect (H$_{0} :\beta _{1} =\beta _{2}$, $\chi ^{2} \left( 1\text {df}\right) =164$, $p <.001$). The within-person effect indicates that, on average, a higher number of daily stressors correspond to a relatively low daily mean positive affect score, and the between-person effect indicates that a higher mean number of daily stressors corresponds to a relatively low daily mean positive affect score. Conditional on these effects, greater daily variation in affect scores corresponds to a higher number of daily stressors ($\hat{\tau }_{1} =0.30$, $s e =0.049$), as well as a higher mean number of daily stressors ($\hat{\tau }_{2} =0.78$, $s e =0.15$), with the latter of the two effects being the largest (H$_{0} :\tau _{1} =\tau _{2}$, $\chi ^{2} \left( 1\text {df}\right) =7.5$, $p =.006$). Additionally, the variance of the random intercept is positively related to the mean number of daily stressors ($\hat{\alpha }_{1} =0.36$, $s e =0.070$), such that the between-subject variation in mean positive affect scores increases with an increase in the mean number of daily stressors. That is, individuals increasingly differ from each other with an increase in the mean number of daily stressors. Overall, it seems that between-subject differences, relative to within-subject variation, in the number of stressors relates most strongly to the different aspects of daily positive affect.

## The Latent Variable Mixed-Effects Location Scale Model

The mixed-effects location scale model is a two-level model for observations nested within individuals (Hedeker et al. 2008). The responses are assumed to be free of measurement error. To address measurement error, a second-order model is developed here. In a second-order version of the model, the repeated measure is a latent variable. That is, at each occasion, a set of manifest variables is assumed to reflect a latent variable that varies by time and person. The subject-specific model is then applied to the repeated measures of the latent variable. Let $\mathbf {y}_{t i}$ be a set of $m_{t i}$ indicators of the latent variable $\eta _{t i}$ observed at occasion *t* for individual *i*. Across $T_{i}$ occasions, let $\mathbf {y}_{i} =\left( \mathbf {y}_{1 i} ,\ldots ,\mathbf {y}_{T_{i}}\right) ^{ \prime }$ and $M_{i}$ denote the number of observations in $\mathbf {y}_{i}$ across indicators and occasions. It is not necessary that each measurement occasion have the same set of indicator variables to represent the latent variable, as long as all share a common indicator that anchors the latent variable over time (Bollen and Curran 2006), and data across occasions need not be complete. Missing data are assumed to be missing at random. A longitudinal measurement model for $\mathbf {y}_{i}$ is

16 $$\begin{aligned} \mathbf {y}_{i} =\mathbf {\tau }_{i} +\mathbf {\Lambda }_{i} (\mathbf {\lambda }) \mathbf {\eta }_{i} +\mathbf {\delta }_{i} , \end{aligned}$$

where $\mathbf {\tau }_{i}$ is an $M_{i}$ x 1 intercept vector, $\mathbf {\Lambda }_{i} (\mathbf {\lambda })$ is an $M_{i}$ *x* *Q* matrix of factor loadings, $\mathbf {\eta }_{i}$ is a *Q* x 1 latent variable vector observed according to $T_{i}$, and $\mathbf {\delta }_{i}$ is an $M_{i}$ x 1 unique item factor vector. The subscript *i* used for both $\mathbf {\tau }_{i}$ and $\mathbf {\Lambda }_{i}$ indicates that both may vary by *i* with regard to their dimensions but not otherwise, whereas both the values and dimensions of $\mathbf {\eta }_{i}$ and $\mathbf {\delta }_{i}$ may vary by *i*. The latent variable $\eta _{t i}$ is assumed to follow a mixed-effects location scale model:

17 $$\begin{aligned} \eta _{t i} =\mathbf {x}_{t i}^{ \prime } (\mathbf {\beta }_{x} +\mathbf {\upsilon }_{i}) +\mathbf {w}_{i}^{ \prime } \mathbf {\beta }_{w} +\varepsilon _{t i} , \end{aligned}$$

where $\mathbf {x}_{t i}$ is a *p* x 1 vector of within-person covariates that would usually include a "1" for the intercept of the model, and $\mathbf {w}_{i}$ is a *q* x 1 vector of between-subject covariates. The coefficient $\mathbf {\beta }_{x}$ contains *p* fixed coefficients linking $\mathbf {x}_{t i}$ to $\eta _{t i}$; $\mathbf {\upsilon }_{i}$ is a set of $p^{ *} \le p$ random subject effects that correspond to one or more of the fixed effects in $\mathbf {\beta }_{x}$. The coefficient $\mathbf {\beta }_{w}$ contains *q* fixed coefficients linking $\mathbf {w}_{i}$ to $\eta _{t i}$. The random effect $\mathbf {\upsilon }_{i}$ is assumed to be independent and normally distributed across individuals as

18 $$\begin{aligned} \mathbf {\upsilon }_{i} \sim N \left( \mathbf {0} ,\mathbf {\Phi }_{\upsilon } \left( \mathbf {w}_{i} ,\mathbf {\alpha } ,\mathbf {\rho }_{\upsilon }\right) \right) , \end{aligned}$$

where $\mathbf {\Phi }_{\upsilon }$ is a function of $\mathbf {w}_{i}$, $\mathbf {\alpha } =\left( \alpha _{0} ,\ldots ,\alpha _{q}\right) \text {,}$ and $\mathbf {\rho }_{\upsilon } =\left( \rho _{1} ,\ldots \right)$. The residual $\mathbf {\varepsilon }_{i}$, specific to the individual with elements specific to the occasions, is assumed to be normally distributed:

19 $$\begin{aligned} \mathbf {\varepsilon }_{i} \sim N \left( \mathbf {0} ,\mathbf {\Theta }_{\varepsilon } \left( \mathbf {x}_{t i} ,\mathbf {w}_{i} ,\mathbf {\tau }\text {,}a_{i}\right) \right) , \end{aligned}$$

where $\mathbf {\Theta }_{\varepsilon }$ is a function of $\mathbf {x}_{t i}$, $\mathbf {w}_{i}$, $\mathbf {\tau }$, and $a_{i}$, with $\mathbf {\tau } =\left( \mathbf {\tau }_{x} ,\mathbf {\tau }_{w}\right)$ similar to (4) in which the coefficients are the effects of within- and between-subject covariates, respectively. Assuming the residuals are independent between occasions, then $\mathbf {\Theta }_{\varepsilon } =\sigma _{\varepsilon }^{2} \mathbf {I}_{n_{i}}$, where $\sigma _{t i}^{2}$ is a function of $\mathbf {x}_{t i}$ and $\mathbf {w}_{i}$ with random effect $a_{i}$, and where $a_{i}$ is assumed to be normally distributed: $a_{i} \sim N \left( 0 ,\phi _{a}^{2}\right) \text {.}$ Thus, the model for the observed response $\mathbf {y}_{i}$ is

20 $$\begin{aligned} \mathbf {y}_{i} =\mathbf {\tau }_{i} +\mathbf {\Lambda }_{i} (\mathbf {\lambda }) \left( \mathbf {\Gamma }_{i} \left( \mathbf {x}_{t i} ,\mathbf {w}_{i}\right) \mathbf {\beta }_{i} +\mathbf {\varepsilon }_{i}\right) +\mathbf {\delta }_{i} . \end{aligned}$$

where $\mathbf {\Gamma }_{i} \left( \mathbf {x}_{t i} ,\mathbf {w}_{i}\right)$ is a design matrix containing the within- and between-subject covariates, and $\mathbf {\beta }_{i} =\left( \left( \mathbf {\beta }_{x} +\mathbf {\upsilon }_{i}\right) ^{ \prime } ,\mathbf {\beta }_{w}^{ \prime }\right) ^{ \prime }$ is a stacked set of coefficients linking the within- and between-subject covariates, respectively, to $\mathbf {y}_{i}$. The residual $\mathbf {\delta }_{i}$ is assumed to be normally distributed:

21 $$\begin{aligned} \mathbf {\delta }_{i} \sim N \left( \mathbf {0} ,\mathbf {\Psi }_{i}\right) , \end{aligned}$$

where $\mathbf {\Psi }_{i}$ is a diagonal covariance matrix of the unique item factors.

Similar to the model for manifest variables, the random effect vector $\mathbf {\upsilon }_{i}$ of (18) and the random effect $a_{i}$ in (19) may covary. To allow this, $\mathbf {\upsilon }_{i}$ and $a_{i}$ are stacked in a vector defined by $\mathbf {c}_{i} \left( r\text {x}1\right) =\left( \mathbf {\upsilon }_{i}^{ \prime } ,a_{i}\right) ^{ \prime }$ that includes all $r =p^{ *} +1$ random effects. The joint distribution of $\mathbf {c}_{i}$ is assumed to be

22 $$\begin{aligned} \mathbf {c}_{i} \sim N \left( \mathbf {\mu }_{c} ,\mathbf {\Phi }_{c}\right) , \end{aligned}$$

where the mean vector and covariance matrix are

23 $$\begin{aligned} \mathbf {\mu }_{c} =\left( \begin{array}{c}\mathbf {\beta }_{x} \\ 0\end{array}\right) \end{aligned}$$

and

24 $$\begin{aligned} \mathbf {\Phi }_{c} =\left( \begin{array}{cc}\mathbf {\Phi }_{\upsilon } \left( \mathbf {w}_{i} ,\mathbf {\alpha } ,\mathbf {\rho }_{\upsilon }\right) &{} \mathbf {\phi }_{\upsilon a} \\ \mathbf {\phi }_{\upsilon a}^{ \prime } &{} \phi _{a}^{2}\end{array}\right) , \end{aligned}$$

respectively. Corresponding to this, the probability distribution of $\mathbf {c}_{i}$ is

25 $$\begin{aligned} h_{c} \left( \mathbf {c}_{i}\right) =2 \pi ^{r/2} \left| \mathbf {\Phi }_{c}\right| ^{ -1/2} \exp \left( \left( \mathbf {c}_{i} -\mathbf {\mu }_{c}\right) ^{ \prime } \mathbf {\Phi }_{c}^{ -1} \left( \mathbf {c}_{i} -\mathbf {\mu }_{c}\right) \right) . \end{aligned}$$

Conditional on the random effects in $\mathbf {c}_{i}$, the distribution of $\mathbf {y}_{i}$ is

26 $$\begin{aligned} \mathbf {y}_{i}\vert \mathbf {c}_{i} \sim N \left( \mathbf {\mu }_{y\vert c} ,\mathbf {\Sigma }_{y\vert c}\right) , \end{aligned}$$

where

27 $$\begin{aligned} \mathbf {\mu }_{y\vert c} =\mathbf {\tau }_{i} +\mathbf {\Lambda }_{i} \mathbf {\Gamma }_{i} \mathbf {\beta } \end{aligned}$$

and

28 $$\begin{aligned} \mathbf {\Sigma }_{y\vert c} =\mathbf {\Lambda }_{i} \left( \mathbf {\Gamma }_{i} \mathbf {\Phi }_{\upsilon } \mathbf {\Gamma }_{i}^{ \prime } +\mathbf {\Theta }_{\mathbf {\varepsilon }}\right) \mathbf {\Lambda }_{i}^{ \prime } +\mathbf {\Psi }_{i} , \end{aligned}$$

where $\mathbf {\beta } =\left( \mathbf {\beta }_{x}^{ \prime } ,\mathbf {\beta }_{w}^{ \prime }\right) ^{ \prime }$ , $\mathbf {\Gamma }_{i} =\mathbf {\Gamma }_{i} \left( \mathbf {x}_{t i} ,\mathbf {w}_{i}\right)$, $\mathbf {\Phi }_{\upsilon } =\mathbf {\Phi }_{\upsilon } \left( \mathbf {w}_{i} ,\mathbf {\alpha } ,\mathbf {\rho }_{\upsilon }\right)$, $\mathbf {\Theta }_{\mathbf {\varepsilon }} =\mathbf {\Theta }_{\mathbf {\varepsilon }} \left( \mathbf {x}_{t i} ,\mathbf {w}_{i} ,\mathbf {\tau } ,a_{i}\right)$, and $\mathbf {\Psi }_{i} =\mathbf {\Psi }_{i} \left( \mathbf {\psi }\right)$. The conditional probability density function is defined as

29 $$\begin{aligned} h_{y\vert c} \left( \mathbf {y}_{i}\vert a_{i}\right) =2 \pi ^{ -p/2} \left| \mathbf {\Sigma }_{y\vert c}\right| ^{ -1/2} \exp \left( \left( \mathbf {y}_{i} -\mathbf {\mu }_{y\vert c}\right) ^{ \prime } \mathbf {\Sigma }_{y\vert c}^{ -1} \left( \mathbf {y}_{i} -\mathbf {\mu }_{y\vert c}\right) \right) . \end{aligned}$$

The marginal distribution of $\mathbf {y}_{i}$ is then

30 $$\begin{aligned} g \left( \mathbf {y}_{i}\right) ={\displaystyle \int _{\mathbf {c}_{i}}}h_{y} \left( \mathbf {y}_{i}\vert \mathbf {c}_{i}\right) h_{c} \left( \mathbf {c}_{i}\right) d \mathbf {c}_{i} . \end{aligned}$$

The parameters of the model are $\mathbf {\gamma } =\left( \mathbf {\beta } ,\mathbf {\alpha } ,\mathbf {\rho }_{\upsilon } ,\mathbf {\tau } ,\mathbf {\phi }_{\upsilon } ,\mathbf {\phi }_{\upsilon a}^{{}} ,\phi _{a}^{2} ,\mathbf {\psi }\right)$. Given a sample of observations, $\mathbf {y}_{1} ,\ldots ,\mathbf {y}_{N}$, the marginal log-likelihood of $\mathbf {\gamma }$ is

31 $$\begin{aligned} \text {ln}L \left( \mathbf {\gamma }\right) ={\displaystyle \sum _{i =1}^{N}}\ln g \left( \mathbf {y}_{i}\right) . \end{aligned}$$

Due to the random effect $a_{i}$, the model for $\mathbf {y}_{i}$ is nonlinear in one of the random effects, and consequently, the integral in (30) does not have a closed-form expression. Steps taken to estimate the integral are described in the next section.

### Simplifying the Likelihood Function

The latent variable mixed-effects location scale model is more complicated than the version of the model for manifest variables. This is due to the measurement model needed for the set of response variables at multiple occasions, in addition to the random effects (one of which enters the model in a nonlinear manner) that are used in the model for the repeated measures of the latent variable. Interestingly, it is possible to simplify the calculation of the $i^{t h}$ individual’s contribution to the likelihood function by analytically eliminating the random effect $\mathbf {\upsilon }_{i}$ from the marginal distribution in (30). This can also be done for the marginal distribution in (14) based on the model for manifest variables (see Appendix A). Based on the fact that, conditional on $a_{i}$, the model is linear in $\mathbf {\upsilon }_{i}$, only the conditional distribution given $a_{i}$ is needed (cf: du Toit and Cudeck 2009). Because the random effects contained in $\mathbf {c}_{i}$ are jointly normally distributed, the conditional distribution of $\mathbf {\upsilon }_{i}$ given $a_{i}$ is also normal,

32 $$\begin{aligned} \mathbf {\upsilon }_{i}\vert a_{i} \sim N \left( \mathbf {\mu }_{\beta .a} ,\mathbf {\Phi }_{\beta .a}\right) , \end{aligned}$$

with mean vector and covariance matrix (Morrison 1990, 3rd ed., p. 92)

33 $$\begin{aligned} \mathbf {\mu }_{\beta .a} =\mathbf {\beta } +\frac{\mathbf {\phi }_{\upsilon a}}{\phi _{a}^{2}} a_{i} \end{aligned}$$

and

34 $$\begin{aligned} \mathbf {\Phi }_{\upsilon .a} =\mathbf {\Phi }_{\upsilon } \left( \mathbf {w}_{i} ,\mathbf {\alpha } ,\mathbf {\rho }_{\upsilon }\right) -\mathbf {\phi }_{\upsilon a} (\phi _{a}^{2})^{ -1} \mathbf {\phi }_{\upsilon a}^{ \prime } , \end{aligned}$$

respectively. It follows that the distribution of $\mathbf {y}_{i}$ conditional on $a_{i}$ is normal:

35 $$\begin{aligned} \mathbf {y}_{i}\vert a_{i} \sim N \left( \mathbf {\mu }_{y .a} ,\mathbf {\Sigma }_{y .a}\right) , \end{aligned}$$

with

36 $$\begin{aligned} \mathbf {\mu }_{y .a} =\mathbf {\tau }_{i} +\mathbf {\Lambda }_{i} \mathbf {\Gamma }_{i} \left( \mathbf {x}_{t i} ,\mathbf {w}_{i}\right) \mathbf {\mu }_{\beta .a} \end{aligned}$$

and

37 $$\begin{aligned} \mathbf {\Sigma }_{y .a} =\mathbf {\Lambda }_{i} \left( \mathbf {\Gamma }_{i} \left( \mathbf {x}_{t i} ,\mathbf {w}_{i}\right) \mathbf {\Phi }_{\upsilon .a} \mathbf {\Gamma }_{i} \left( \mathbf {x}_{t i} ,\mathbf {w}_{i}\right) ^{ \prime } +\mathbf {\Theta }_{\varepsilon }^{a}\right) \mathbf {\Lambda }_{i}^{ \prime } +\mathbf {\Psi }_{i}\text {.} \end{aligned}$$

Assuming independence between the residuals relating to the latent variable between occasions,

38 $$\begin{aligned} \mathbf {\Theta }_{\varepsilon }^{a} =\mathbf {I}_{n_{i}}\exp \left\{ \tau _{0} +\tau _{x 1} +\ldots +\tau _{x p} +\ldots +\tau _{w 1} +\ldots +\tau _{w q} +a_{i}\right\} . \end{aligned}$$

The probability density function of $\mathbf {y}_{i}$ given $a_{i}$ is

39 $$\begin{aligned} h_{y\vert a} \left( \mathbf {y}_{i}\vert a_{i}\right) =2 \pi ^{ -p/2} \left| \mathbf {\Sigma }_{y .a}\right| ^{ -1/2} \exp \left( \left( \mathbf {y}_{i} -\mathbf {\mu }_{y .a}\right) ^{ \prime } \mathbf {\Sigma }_{y .a}^{ -1} \left( \mathbf {y}_{i} -\mathbf {\mu }_{y .a}\right) \right) . \end{aligned}$$

The marginal distribution of $\mathbf {y}_{i}$ given $a_{i}$ is then

40 $$\begin{aligned} g \left( \mathbf {y}_{i}\right) ={\displaystyle \int _{a_{i}}}h_{y\vert a} \left( \mathbf {y}_{i}\vert a_{i}\right) h_{a} \left( a_{i}\right) d a_{i} , \end{aligned}$$

thus requiring a one-dimensional integral. The log-likelihood function is then

41 $$\begin{aligned} \text {ln}L \left( \mathbf {\gamma }\right) ={\displaystyle \sum _{i =1}^{N}}\ln g \left( \mathbf {y}_{i}\right) , \end{aligned}$$

where $\mathbf {\gamma } =\left( \mathbf {\beta } ,\mathbf {\alpha } ,\mathbf {\rho }_{\upsilon } ,\mathbf {\tau } ,\mathbf {\phi }_{\upsilon } ,\mathbf {\phi }_{\upsilon a}^{{}} ,\phi _{a}^{2} ,\mathbf {\psi }\right)$. Again, as the model for $\mathbf {y}_{i}$ is nonlinear in $a_{i}$, the integral in (40) does not have a closed-form expression, and so estimation relies on an approximation. In the empirical example presented in the next section, Gauss–Hermite quadrature is used to evaluate the integral. A computer program, such as one written with SAS IML, may be used to carry out estimation.

### Example: Daily Latent Positive Affect

One shortcoming of the previous analysis of the daily mean positive affect scores was the fact that the measurement error of the manifest variables was not addressed. Here, a latent variable mixed-effects location scale model is applied to the item-level response data. Measurement invariance is generally required for a meaningful interpretation of a latent variable that is studied over repeated occasions because it is necessary that an instrument perform in the same manner regardless of the measurement occasion (Chan, 1998; Meredith 1993; Millsap 2011). Assuming a single dimension for the set of item responses, measurement invariance across the 8 days was evaluated. First, equality of factor loadings was tested, followed by similar tests about the item intercepts and then the uniqueness. Relative to a model without restrictions placed on the factor loadings, item intercepts or uniquenesses (Deviance = 31,009, BIC = 32,759), LRTs suggested equal factor loadings by item across days ($\chi ^{2} \left( 28 d f\right) =35.4\text {,}$ $p =.16$; Deviance = 31,045, BIC = 32,625), equal item intercepts by item across days (with the exception of item 4 on day 8) ($\chi ^{2} \left( 27 d f\right) =36.8\text {,}$ $p =.10$, Deviance = 31,046, BIC = 32,632), and both equal factor loadings and item intercepts by item across days (with the exception of allowing for unique intercepts for item 10 on days 1 and 8 and item 11 day 8) ($\chi ^{2} \left( 53 d f\right) =62.47\text {,}$ $p =.18$, Deviance = 31,072, BIC = 32,500). Although the BIC was lowest for a model that additionally constrained the uniquenesses to be equal by item and across days (Deviance = 31,226, BIC = 32,441), a LRT did not support this ($\chi ^{2} \left( 35 d f\right) =154.03\text {,}$ $p <.001$). The analysis proceeded assuming equal uniquenesses, along with equal factor loadings and item intercepts, by item across days, with the results evaluated for the tenability of this assumption.

A latent variable model based on the daily responses to the 5-item set assumed that the intercept of the first item equation was equal to 0 and that the factor loading was equal to 1:

$$\begin{aligned}\left[ \begin{array}{c}y_{1 1 i} \\ y_{2 1 i} \\ y_{3 1 i} \\ y_{4 1 i} \\ y_{5 1 i} \\ \vdots \\ y_{1 8 i} \\ y_{2 8 i} \\ y_{3 8 i} \\ y_{4 8 i} \\ y_{5 8 i}\end{array}\right] =\left[ \begin{array}{c}0 \\ \tau _{2} \\ \tau _{3} \\ \tau _{4} \\ \tau _{5} \\ \vdots \\ 0 \\ \tau _{2} \\ \tau _{3} \\ \tau _{4} \\ \tau _{5}\end{array}\right] +\left[ \begin{array}{cccc}1 &{} 0 &{} \cdots &{} 0 \\ \lambda _{2} &{} 0 &{} \cdots &{} 0 \\ \lambda _{3} &{} 0 &{} \cdots &{} 0 \\ \lambda _{4} &{} 0 &{} \cdots &{} 0 \\ \lambda _{5} &{} 0 &{} \cdots &{} 0 \\ \vdots &{} \vdots &{} \ddots &{} \vdots \\ 0 &{} 0 &{} \cdots &{} 1 \\ 0 &{} 0 &{} \cdots &{} \lambda _{2} \\ 0 &{} 0 &{} \cdots &{} \lambda _{3} \\ 0 &{} 0 &{} \cdots &{} \lambda _{4} \\ 0 &{} 0 &{} \cdots &{} \lambda _{5}\end{array}\right] \left[ \begin{array}{c}\eta _{1 i} \\ \vdots \\ \eta _{8 i}\end{array}\right] +\left[ \begin{array}{c}\delta _{1 1 i} \\ \delta _{2 1 i} \\ \delta _{3 1 i} \\ \delta _{4 1 i} \\ \delta _{5 1 i} \\ \vdots \\ \delta _{1 8 i} \\ \delta _{2 8 i} \\ \delta _{3 8 i} \\ \delta _{4 8 i} \\ \delta _{5 8 i}\end{array}\right] \text {.} \end{aligned}$$

Elements of the unique factor score vector $\mathbf {\delta }_{i} =\left( \delta _{1 1 i} ,\ldots ,\delta _{5 8 i}\right)$ were assumed to be independently and normally distributed across individuals as $\mathbf {\delta }_{i} \sim N \left( \mathbf {0} ,\mathbf {\Psi }\right)$, with scores assumed to be independent between days with constant variance across days. Let $\mathbf {\tau } =\left( 0 ,\tau _{2} ,\tau _{3} ,\tau _{4} ,\tau _{5}\right)$ , $\mathbf {\lambda } =\left( 1 ,\lambda _{2} ,\lambda _{3} ,\lambda _{4} ,\lambda _{5}\right)$, and $\mathbf {\psi } =\left( \psi _{1} ,\psi _{2} ,\psi _{3} ,\psi _{4} ,\psi _{5}\right)$, where $\mathbf {\psi }$ is the vector of the unique factor score variances relating to the five scale items. To then fit a latent variable mixed-effects location scale model, the latent measure of positive affect was assumed to follow a mixed-effects location scale model. Similar to the daily scores based on averages of the item responses, the latent measure of daily positive affect was expected to fluctuate across days at the individual level and so was assumed to follow a random intercept model:

$$\begin{aligned}\eta _{t i} =\beta _{0 i} +\varepsilon _{t i}\text {,} \end{aligned}$$

where, for participant *i*, $\beta _{0 i}$ is the latent measure of positive affect on day *t*, and $\varepsilon _{t i}$ is the discrepancy between the latent score and the fitted value on day *t*. The random intercept is assumed to be normally distributed:

$$\begin{aligned}\beta _{0 i} \sim N \left( \beta _{0} ,\phi _{\upsilon }^{2}\right) \text {,} \end{aligned}$$

where $\beta _{0}$ is the expected latent response across days, and $\phi _{\upsilon }^{2} =\exp \left\{ \alpha _{0}\right\} \text {.}$ At the occasion level, the residual is assumed to be normally distributed:

$$\begin{aligned} \varepsilon _{t i} \sim N \left( 0 ,\mathbf {I} \sigma _{\varepsilon }^{2}\right) \text {,} \end{aligned}$$

where $\sigma _{\varepsilon }^{2} =\exp \left\{ \tau _{0} +a_{i}\right\} \text {,}$and $a_{i}$ is a random subject effect that is assumed to be normally distributed:

$$\begin{aligned}a_{i} \sim N \left( 0 ,\phi _{a}^{2}\right) \text {.} \end{aligned}$$

At the subject level, the random intercept and the random effect of the within-subject residual variance may covary: $\phi _{\upsilon a} =c o v \left( \upsilon _{i} ,a_{i}\right)$. The model, henceforth referred to as Model B$_{2}$, has five parameters of central interest, along with the parameters relating to the measurement model of *y*: $\mathbf {\gamma } =\left( \mathbf {\tau } ,\mathbf {\lambda } ,\mathbf {\psi } ,\beta _{0} ,\alpha _{0} ,\tau _{0} ,\phi _{a}^{2} ,\phi _{\upsilon a}\right) \text {.}$ Code developed for use with SAS IML for estimation of the model is provided in Appendix B.

**Table 2:** ML estimates of a latent variable mixed-effects location scale model for daily positive affect ($n=435$).

Measurement	Model B$_{1}$	Model B$_{2}$	Model B$_{3}$	Model B$_{4}$
$\tau _{2}$	$-0.59(0.056)$	$-0.58(0.056)$	$-0.58\left( 0.055\right)$	$-0.58\left( 0.056\right)$
$\tau _{3}$	$-0.63(0.066)$	$-0.63(0.066)$	$-0.61\left( 0.064\right)$	$-0.63\left( 0.065\right)$
$\tau _{4}$	$-0.54(0.059)$	$-0.56(0.059)$	$-0.55\left( 0.058\right)$	$-0.57\left( 0.059\right)$
$\tau _{5}$	$-1.4(0.079)$	$-1.4(0.078)$	$-1.4\left( 0.077\right)$	$-1.4\left( 0.078\right)$
$\lambda _{2}$	1.15(0.018)	1.15(0.018)	$1.15\left( 0.018\right)$	$1.15\left( 0.018\right)$
$\lambda _{3}$	1.13(0.022)	1.13(0.022)	$1.13\left( 0.021\right)$	$1.14\left( 0.021\right)$
$\lambda _{4}$	1.15(0.019)	1.15(0.020)	$1.15\left( 0.019\right)$	$1.15\left( 0.019\right)$
$\lambda _{5}$	1.32(0.026)	1.32(0.026)	$1.32\left( 0.025\right)$	$1.32\left( 0.026\right)$
$\psi _{1}$	0.20(0.006)	0.20(0.006)	$^{a}$	$^{a}$
$\psi _{2}$	0.22(0.007)	0.22(0.007)	$^{a}$	$^{a}$
$\psi _{3}$	0.35(0.010)	0.35(0.010)	$^{a}$	$^{a}$
$\psi _{4}$	0.22(0.007)	0.21(0.007)	$^{a}$	$^{a}$
$\psi _{5}$	0.53(0.015)	0.52(0.015)	$^{a}$	$^{a}$
*Mean structure*				
$\beta _{0},$ intercept	2.99(0.028)	2.99(0.028)	$2.99\left( 0.028\right)$	3.20(0.042)
$\beta _{1},$ DS$_{ti}^{*}$				$-0.10(0.011)$
$\beta _{2},$ DMS$_{i}$				$-0.38(0.060)$
*Covariance Structure*				
*Between-subject*				
$\alpha _{0}$, intercept	$-2.41(0.15)$	$-1.21(0.077)$	$-1.21\left( 0.077\right)$	$-1.21(0.11)$
$\alpha _{1},$ DMS$_{i}$				$-0.20(0.17)$
$\phi _{a}$		1.37(0.081)	$1.40\left( 0.081\right)$	1.29(0.079)
$\rho _{a\beta _{0i}}$		$-0.43(0.054)$	$-0.43\left( 0.053\right)$	$-0.40(0.056)$
*Within-Subject*				
$\tau _{0}$, intercept	$-1.93(0.041)$	$-2.66(0.093)$	$-2.69\left( .095\right)$	$-3.20(0.13)$
$\tau _{1},$DS$_{ti}^{*}$				0.30(0.071)
$\tau _{2},$DMS$_{i}$				0.89(0.17)
$-2lnl$	33678	33034	32864	32711
AIC	33710	33070	32970	32827

Notes: ML estimation was carried out using Gauss–Hermite quadrature with 10 quadrature points to evaluate the integral. $^{a}$ ML estimates are given in Appendix C

ML estimates of Model B$_{2}$ are given in Table 2. The values presented in the table are based on an analysis carried out using a trust-region optimization method (Moré and Sorensen 1983) available through SAS IML and used to optimize the likelihood function. The solution was evaluated using quadrature points that ranged from 5 to 30 with increments of 5 points, resulting in no appreciable differences in the parameter estimates or standard errors when using the different number of quadrature points. The estimates provided in Table 2 are based 10 quadrature points. To obtain starting values for estimation, results from the factor analysis model supplied values for the measurement model. A fixed-effects model was then fit, followed by adding a random intercept, then a random effect in the within-subject residual variance model with its covariance with the random intercept. To assess whether there was appreciable heterogeneity of variance in the occasion-level residuals, estimates were obtained for a model (Model B$_{1}$) that assumed $\phi _{a}^{2} =0$ and compared to those under the model that assumed $\phi _{a}^{2} >0$ (Model B$_{2}$). The model assuming $\phi _{a}^{2} >0$ results in an improvement in model fit in terms of the AIC, from 33,710 down to 33,070, suggesting heterogeneity of the within-person residual variance. A LRT of the goodness-of-fit between the two models using 2 df is $\chi _{2}^{2} =33,678 -33,034 =644\text {,}$ $p <.001\text {,}$ supporting the need for the random effect in the residual variance model. As noted earlier, the assumption that the uniquenesses for each item were equal across the days of measurement was not tenable according to a LRT. Thus, Model B$_{2}$ was fit relaxing the assumption that the uniquenesses were equal, with this model henceforth called Model B$_{3}$. Estimates from this model are given in Table 2 (with additional estimates provided in Appendix C). A LRT comparing Model B$_{2}$ and Model B$_{3}$ supports the need to allow for the unique values. From the estimates based on Model B$_{3}$, the estimated correlation between the random intercept and the random subject effect for the within-subject residual variance is $r = -.43$, an increase from $r = -.36$ in the model that relied on mean scores, but again suggesting a tendency for those with a relatively low daily variation to also have a relatively high mean positive affect score across days. Similar to the analysis that relied on the means of the item response variables, the negative correlation suggests a tendency for those who have a relatively high mean level of positive affect across days to also have a relatively low degree of day-to-day variability. Thus, individuals who had higher estimated averages in affect across days also showed greater stability in their affect across days. Again, two possible conclusions from this are that those with greater overall positive affect are more stable in their daily positive affect or there is a ceiling effect of measurement. Available in supplementary materials are empirical Bayes estimates of the location and scale values based on Model B$_{3}$, along with estimated conditional residuals between the observed item scores and the fitted values based on the ML estimates from fitting Model B$_{3}$.

In the previous analysis that relied on sample mean scores, it was not possible to estimate the state residual variance of positive affect scores without the measurement error naturally inherent in manifest scores. In the latent variable model that accounts for measurement error, the estimated state residual variance, assuming homogeneity of the within-person residual variance of daily latent scores, is $\exp \left\{ -1.93\right\} =0.15$, and the estimated trait variance is $\exp \left\{ -2.41\right\} =0.090$, with the state variance almost double the size of the trait variance. With a relatively high residual state variance, variation in positive affect seems to be due more to daily variability rather than between-person differences. Relaxing the assumption of homogeneity of the within-person residual variance, however, gives a different description of the data. First, given the improvement in model fit, it does not seem reasonable to conclude that the residual state variance is common across individuals, but rather, that it varies according to person. The estimated residual state variance for the typical individual is $\exp \left\{ \tau _{0} +.5 \phi _{a}^{2}\right\}$ (Hedeker et al. 2008) $=\exp \left\{ -2.56 +.5 \left( 1.32\right) ^{2}\right\} =0.18$ and the estimated trait variance is $\exp \left\{ -1.21\right\} =0.30$, with the trait variance now being greater than the magnitude of the residual state variance, thus suggesting that, for the typical individual, variation in positive affect scores is more highly attributable to between-person differences than within.

As discussed earlier, applications of a mixed-effects location scale model generally do not account for measurement error in response data. If scores include measurement error, the residuals reflect a combination of within-subject variation both about the fitted model and that due to measurement error. To evaluate the impact of partitioning the variance of the manifest scores as can be done using a latent variable model, a version of Model B$_{3}$ was fit in which a single variance was used to represent these combined sources. The factor loadings, item intercepts, factor mean, standard deviation of $a_{i}$ and correlation between $a_{i}$ and $\upsilon _{i}$ were set equal to the estimates resulting from fitting Models B$_{3}$, resulting in an estimated combined common variance of $\exp \left\{ -0.77\right\} =0.46$. From Model B$_{3}$ that partitions the variance due to measurement error and variation due to the fitted model, the common variance at the occasion level is estimated as $\exp \left\{ -2.56\right\} =0.08$, a substantial decrease from that in which the two sources of variation are combined.

#### Daily Stressors

To study the within- and between-subject effects of daily stressors on latent positive affect, the person-mean centered and mean number of daily stressors were included in the latent variable location scale model, similar to what had been done when evaluating positive affect using the manifest scores directly:

$$\begin{aligned}\eta _{t i} =\beta _{0 i} +\beta _{1} \text {\textit{DS}}_{t i}^{ *} +\beta _{2} \text {\textit{DS}}_{ .i} +\varepsilon _{t i}\text {.} \end{aligned}$$

Additionally, the two measures of daily stressors were included in the model of the occasion-level residual variance relating to the latent affect measures:

$$\begin{aligned} \sigma _{\varepsilon }^{2} =\exp \left\{ \tau _{0} +\tau _{1} \text {\textit{DS}}_{t i}^{ *} +\tau _{2} \text {\textit{DS}}_{ .i} +a_{i}\right\} \text {,} \end{aligned}$$

and the individual mean number of stressors was included in the variance model of the random intercept:

$$\begin{aligned}\phi _{\upsilon }^{2} =\exp \left\{ \alpha _{0} +\alpha _{1} \text {\textit{DS}}_{ .i}\right\} \text {.} \end{aligned}$$

Starting values for the effects of covariates were set to 0. Estimates of this model are in the last column of Table 2. For those with no reported daily stressors, the point estimate of the overall positive affect score across days and individuals is $\hat{\beta }_{0} =3.20$ ($s e =0.13$), a value that is higher than the point estimate of 3.05 obtained from the model that relied on the mean of the manifest variables. For daily positive affect, the estimates suggest an overall negative within-person ($\hat{\beta }_{1} = -0.10$, $s e =0.011$), as well as between-person ($\hat{\beta }_{2} = -0.38$, $s e =0.060$), effect of the number of daily stressors on daily mean positive affect. Thus, on average, the within-person effect indicates that a higher number of daily stressors tends to correspond to a lower daily positive affect level, and the between-person effect indicates that a higher mean number of daily stressors across days tends to correspond to a lower daily mean positive affect, with the latter being greater than the former (H$_{0} :\beta _{1} =\beta _{2}$, $\chi ^{2} \left( 1\text {df}\right) =22$, $p <.001$). In the previous analysis based on sample mean scores to represent positive affect, the relative magnitudes of these two effects were somewhat different relative to the estimates from the latent variable model, but inference from both models in terms of the direction of the effects is the same with the between-subject differences in the number of daily stressors having the greater impact on positive affect. Conditional on these effects, greater daily variation in affect scores corresponds to a high number of daily stressors ($\hat{\tau }_{1} =0.30$, $s e =0.071$), as well as a higher number of mean daily stressors ($\hat{\tau }_{2} =0.89$, $s e =0.17)\text {,}$, with the latter being greater than the former (H$_{0} :\tau _{1} =\tau _{2}$, $\chi ^{2} \left( 1\text {df}\right) =9.7$, $p =.002$). These estimates are comparable to those obtained under the model that relied on manifest scores. Also under the latent variable model, the variance of the random intercept does not have a clear relationship with the mean number of daily stressors ($\hat{\alpha }_{1} = -0.20$, $s e =0.17$), unlike the result under the standard model where it seemed that individuals differed to a greater extent from each other as their mean number of daily stressors increased. From this, one might conclude that there is no appreciable association between the between-subject variation in positive affect levels and the mean number of stressors under the latent variable model but that the association is instead strong and positive when studied under the standard version of the model. Finally, a comparison of the estimated standard errors of the two models overall indicates that the precision of the estimates is generally better under the model that used the sample means scores. As the latent variable model is preferred for the sake of accounting for measurement error in the manifest measures, it is possible that the precision of the estimates is negatively biased in the standard version of the model when scores do contain measurement error and are best reflected by those values obtain under the latent variable model.

## Discussion

A mixed-effects location scale model extends the more common applications of latent curve and mixed-effects models to uniquely address heterogeneity of variance both in the random effects at the second level and the within-subject residuals. Some advancements of this framework have included extensions to bivariate versions of the model to study multiple longitudinal variables, including dyad data involving distinguishable individuals (Pugach et al. 2014), a 3-level model (Lin et al. 2018), and joint models to handle combinations of response distributions (Lu 2017). Most applications have relied on ML estimation, but Bayesian estimation of these models is an alternative (Lin et al.). One benefit of fitting a mixed-effects model that includes a model for the variance of a random effect is that the variance can be a function of between-subject covariates. Recalling the example presented in this paper, the variance of the random intercept reflected the magnitude of individual differences in the expected value of positive affect across multiple days. Using a mixed-effects location scale model that was applied to sample mean scores, the variance of the random intercept was studied as a function of the mean number of daily stressors reported by study participants. Based on the model, the between-subject variability in mean positive affect levels increased with an increase in the daily mean number of stressors. Although not considered here, it would also be possible to allow the variance of a random subject effect to also vary according to occasion-specific covariates. Similarly, the assumption of homogeneity of the residual variances may be relaxed by allowing both within- and between-subject covariates to account for variation in the occasion-specific residual variance. Additionally, a random effect may be included in the model to allow for heterogeneity of the occasion-level residuals, even after adjusting for the effects of covariates. $^{1}$ Access to SAS software is available for free to academics (https://www.sas.com/en_us/software/on-demand-for-academics.html).

Applications of a mixed-effects location scale model often involve psychological response data, although the standard application of the model assumes that the response variable is measured without error. This paper aimed to extend the mixed-effects location scale model by specifying a measurement model for repeated measures of a set of indicator variables assumed to reflect an underlying construct. Thus, this approach permits evaluation of the assumption of measurement invariance, and if needed, adjustments to the latent variable mixed-effects location scale model may be made if needed. In the empirical example provided, for instance, the assumption of equality of the item uniquenesses across days was not defensible, and so the model took this into account. Thus, the model developed in this paper included important aspects of a mixed-effects location scale model while addressing measurement error that is inherent to many psychological variables. Thus, a clear benefit to using the latent variable version of the model is that the occasion-level residuals relate to the latent variable, and as such, reflect true within-subject variation.

From the empirical examples provided in this paper, there were appreciable differences between the results from the standard application of the model and those from the latent variable version of the model, with the greatest impact falling on the estimated covariance structure of the model. That is, the differences in the estimates based on the fixed-effects part of the model were only slightly different between models. With regard to the covariance structure, the differences were far more appreciable in terms of the magnitude of covariate effects, and this lead to differences about whether within-subject versus between-subject effects were most important. To better understand important differences between the two versions of the model requires a data simulation study. This would also allow for an examination of other factors, such as the number of repeated measures and the number of study participants needed, for reliable estimation of these models and to understand the biases that may come from either version of the model. Finally, although the empirical examples relied on cases that were complete with respect to the variables chosen for analysis, the model developed in this paper does not require complete data. Missing data are common in longitudinal investigations, and understanding the impact of missing data on the estimation of mixed-effects location scale models is worthy of further study.

### Supplementary Information

Below is the link to the electronic supplementary material.

Supplementary file 1 (docx 38 KB)

Supplementary file 2 (docx 28 KB)

## Appendix A

Under a mixed-effects location scale model, the marginal distribution of the repeated measures can be expressed as a conditional distribution of the response given the nonlinear random effect $a_{i}$ in (3) (cf: du Toit and Cudeck 2009), and thus, the conditional distribution requires an integral equation of only one dimension. First, because the random effects ($\mathbf {\upsilon }_{i} ,a_{i}$) are jointly normally distributed, the conditional distribution of $\mathbf {\upsilon }_{i}$ given $a_{i}$ is also normal:

$$\begin{aligned}\mathbf {\upsilon }_{i}\vert a_{i} \sim N \left( \mathbf {\mu }_{\upsilon .a} ,\mathbf {\Phi }_{\upsilon .a}\right) \text {,} \end{aligned}$$

with mean vector and covariance matrix (Morrison 1990, 3rd ed., p. 92)

$$\begin{aligned}\mathbf {\mu }_{\upsilon .a} =\mathbf {\beta } +\frac{\mathbf {\phi }_{\upsilon a}}{\phi _{a}^{2}} a_{i}\text {,} \end{aligned}$$

and

$$\begin{aligned} \mathbf {\Phi }_{\upsilon .a} =\mathbf {\Phi }_{\upsilon } \left( \mathbf {w}_{i} ,\mathbf {\alpha } ,\mathbf {\rho }_{\upsilon }\right) -\mathbf {\Phi }_{\upsilon a}^{{}} (\phi _{a}^{2})^{ -1} \mathbf {\Phi }_{\upsilon a}^{ \prime }\text {,} \end{aligned}$$

respectively. It follows that the distribution of $\mathbf {y}_{i}$ given $a_{i}$ is normal:

$$\begin{aligned}\mathbf {y}_{i}\vert a_{i} \sim N \left( \mathbf {\mu }_{y .a} ,\mathbf {\Sigma }_{y .a}\right) \text {,} \end{aligned}$$

with

$$\begin{aligned}\mathbf {\mu }_{y .a} =\mathbf {\Gamma }_{i} \left( \mathbf {x}_{t i} ,\mathbf {w}_{i}\right) \mathbf {\mu }_{\upsilon .a} \end{aligned}$$

and

$$\begin{aligned}\mathbf {\Sigma }_{y .a} =\mathbf {\Gamma }_{i} \left( \mathbf {x}_{t i} ,\mathbf {w}_{i}\right) \mathbf {\Phi }_{\upsilon .a} \mathbf {\Gamma } \left( \mathbf {x}_{t i} ,\mathbf {w}_{i}\right) ^{ \prime } +\mathbf {\Theta }_{\varepsilon }^{a}\text {.} \end{aligned}$$

Assuming independence of the residuals between occasions, the within-subject variance is modeled by

$$\begin{aligned}\mathbf {\Theta }_{\varepsilon }^{a} =\mathbf {I}\exp \left\{ \theta _{0} +\mathbf {\tau }_{x} \mathbf {x}_{t i} +\mathbf {\tau }_{w} \mathbf {w}_{i} +a_{i}\right\} \text {.} \end{aligned}$$

Conditional on $a_{i}$, the model in (1) with a random effect included in the within-subject variance model in (3) is linear in $\mathbf {\nu }_{i}$. Consequently, it is possible to analytically eliminate $\mathbf {\nu }_{i}$ from the marginal distribution in (4), and as a result, the calculation is simplified. The conditional distribution of $\mathbf {y}_{i}$ given $a_{i}$ is

$$\begin{aligned}\mathbf {y}_{i}\vert a_{i} \sim N \left( \mathbf {\mu }_{i} ,\mathbf {\Sigma }_{i}\right) \text {,} \end{aligned}$$

where

$$\begin{aligned}\mathbf {\mu }_{i} =\mathbf {\Gamma } \left( \mathbf {x}_{t i} ,\mathbf {w}_{i}\right) \mathbf {\beta } \end{aligned}$$

and

$$\begin{aligned}\mathbf {\Sigma }_{i} =\mathbf {\Gamma } \left( \mathbf {x}_{t i} ,\mathbf {w}_{i}\right) \mathbf {\Phi }_{\upsilon } \left( \mathbf {w}_{i} ,\mathbf {\alpha } ,\mathbf {\rho }_{\upsilon }\right) \mathbf {\Gamma } \left( \mathbf {x}_{t i} ,\mathbf {w}_{i}\right) ^{ \prime } +\mathbf {\Theta }_{\varepsilon } \left( \mathbf {x}_{t i} ,\mathbf {w}_{i} ,\mathbf {\tau }\right) \text {.} \end{aligned}$$

The corresponding probability density function is

$$\begin{aligned}h_{y} \left( \mathbf {y}_{i}\vert a_{i}\right) =2 \pi ^{ -2/N} \left| \mathbf {\Sigma }_{i}\right| ^{ -1/2} \exp \left( -{\frac{1}{2}} \left( \mathbf {y}_{i} -\mathbf {\mu }_{i}\right) ^{ \prime } \mathbf {\Sigma }_{i}^{ -1} \left( \mathbf {y}_{i} -\mathbf {\mu }_{i}\right) \right) \text {.} \end{aligned}$$

The marginal distribution of $\mathbf {y}_{i}$ is then

$$\begin{aligned}g \left( \mathbf {y}_{i}\right) ={\displaystyle \int _{a_{i}}}h_{y} \left( \mathbf {y}_{i}\vert a_{i}\right) h_{a} \left( a_{i}\right) d a_{i}\text {.} \end{aligned}$$

Thus, the marginal distribution of $\mathbf {y}_{i}$ that is an *r* dimensional integral has been reduced to a single dimensional integral. Given a sample of observations, $\mathbf {y}_{1} ,\ldots ,\mathbf {y}_{N}$, the log-likelihood of $\mathbf {\gamma }$ is then

$$\begin{aligned}\text {ln}L \left( \mathbf {\gamma }\right) ={\displaystyle \sum _{i =1}^{N}}\ln g \left( \mathbf {y}_{i}\right) \text {.} \end{aligned}$$

where $$\mathbf {\gamma } =\left( \mathbf {\beta } ,\mathbf {\alpha } ,\mathbf {\rho }_{\mathbf {\upsilon }} ,\mathbf {\tau } ,\mathbf {\phi }_{\upsilon } ,\mathbf {\phi }_{\upsilon a} ,\phi _{a}^{2}\right) \text {.}$$

## Appendix B

The script below is code for estimation of a latent variable mixed-effects model referred to as Model B$_{2}$ in the empirical example. The data file, "daily", is structured in long format with variables given by: id day v y.

proc iml;

use daily;

read all var {id day v y};

* – nodes and weights for 10-point Gauss-Hermite integral approximation of marginal pdf –;

xk = {$$-$$3.4361591188377, $$-$$2.5327316742328, $$-$$1.7566836492999, $$-$$1.0366108297895, −0.3429013272237, 0.3429013272237, 1.0366108297895, 1.7566836492999, 2.5327316742328, 3.4361591188377};

wk = { 7.640432855233e-6, 0.001343645746781, 0.03387439445548, 0.2401386110823, 0.6108626337353, 0.6108626337353, 0.2401386110823, 0.03387439445548, 0.001343645746781, 7.640432855233e-6};

Nq = nrow(xk); * no. of quadrature points;

* – specify aspects specific to the data problem –;

p = 40; * no. of manifest indicators;

Nc = 8; * no. of occasions;

Ns = nrow(y)/p; * no. of subjects;

* – parameter starting values for beta0, alp0, SD(a), rho_va, tau0, lam2-lam5, tau2-tau5, psi1-psi5 –;

th = {2.9 -1.14 1.34 -0.44 -2.59 1.18 1.13 1.15 1.38 -0.72 -0.65 -0.59 -1.5 0.20 0.22 0.40 0.23 0.52 }‘;

Nparm = nrow(th); * – no. of model parameters –;

print " ——————- Number of ———————",,

" Subjects Occasions Parms Quad. pts",,

Ns Nc " " Nparm Nq;

*– design matrix for a random intercept model –;

X = {1 1 1 1 1 1 1 1}‘;

start model(th) global(Ns,Nc,p,id,X,y,Nq,wk,xk);

pie = 3.1415927;

beta = th[1];

alp0 = th[2];

phi00 = sqrt(exp(alp0));

phi = I(2);

phi[2,1] = th[4]; phi=phi+phi‘-I(2);

phi = ((phi00//th[3]) # phi) # (phi00//th[3])‘;

tau0 = th[5];

phi_b_a = phi[1,1] - (phi[1,2] / phi[2,2]) * phi[2,1];

Va = phi[2,2];

XphX = X * phi_b_a * X‘;

lam = I(Nc) @ (1//th[6]//th[7]//th[8]//th[9]);

tau = J(Nc,1,1) @ (0//th[10]//th[11]//th[12]//th[13]);

delta = J(Nc,1,1) @ (th[14]//th[15]//th[16]//th[17]//th[18]); De = diag(delta);

Lnl = 0;

do i = 1 to Ns;

je = i#p;

js = je-p+1;

yi = y[js:je];

Xi = 0;

do k = 1 to Nq;

ax = sqrt(2#Va)#xk[k];

mu_b_a = beta + (phi[1,2] / phi[2,2]) # ax;

mu_y_a = tau + lam * X * mu_b_a;

ei = yi - mu_y_a;

psi = I(Nc) # exp(tau0 + ax);

sig_y_a = lam * (XphX + psi) * lam‘ + De;

siv_y_a = inv(sig_y_a);

Dt = det(sig_y_a);

Xi = Xi + wk[k] # (1/sqrt(Dt)) # exp(-0.5 # (ei‘*siv_y_a*ei));

end;

*– contribution of individual i to loglikelihood –;

Xi = (((2*pie)##(-p/2)) / sqrt(pie)) # Xi;

*– loglikelihood function –;

Lnl = Lnl + log(Xi);

end;      

return(-Lnl);

finish;

* – function optimization –;

call nlptr(rc,thn,’model’,th,{0 1}); thn=thn‘;

vn={’beta0’ ’alp0’ ’SD(a)’ ’rho_va’ ’tau0’ ’lam2’ ’lam3’ ’lam4’ ’lam5’ ’tau2’ ’tau3’ ’tau4’ ’tau5’ ’psi1’ ’psi2’ ’psi3’ ’psi4’ ’psi5’};

* – second-order partial derivatives of the loglikelihood function, evaluated at the solution, for SEs of the estimates –;

call nlpfdd(f,gr,Hs,’model’,thn); gr=gr‘;

* – display estimates with SEs and 95% confidence intervals, gradient and AIC value –;

se = sqrt(vecdiag(inv(Hs)));

me = 2#se; t = thn / se;

print ,,,, th[r=vn f=best6.] " " thn[f=10.4] se[f=10.4] t[c="thn/se" f=10.3] (thn-me)[f=12.4 c="Lb"] (thn+me)[f=10.4 c="Ub"] gr[f=10.4];

AIC = 2#f + 2#nrow(thn); print ,,, " " (f‘)[f=best8. c="LogLike"] " " (2#f)[f=best8. c="Deviance"] " " aic[f=best8.];

quit;

## Appendix C

ML estimates of the uniqueness by item and day under Models B$_{3}$ and B$_{4}$ are given below.

**Table:**

Model B$_{3}$															
Day	1					2					3				
Item	1	2	3	4	5	1	2	3	4	5	1	2	3	4	5
${\hat{\psi }}$	0.20	0.29	0.51	0.31	0.60	0.26	0.25	0.32	0.19	0.43	0.17	0.23	0.33	0.18	0.51
se($\hat{\psi }$)	0.02	0.02	0.04	0.03	0.05	0.02	0.02	0.03	0.02	0.03	0.02	0.02	0.03	0.02	0.04
Day	4					5					6				
Item	1	2	3	4	5	1	2	3	4	5	1	2	3	4	5
${\hat{\psi }}$	0.16	0.20	0.27	0.22	0.58	0.18	0.21	0.29	0.17	0.56	0.21	0.20	0.32	0.19	0.49
se($\hat{\psi }$)	0.01	0.02	0.02	0.02	0.03	0.02	0.02	0.02	0.02	0.04	0.02	0.02	0.03	0.02	0.04
Day	7					8									
Item	1	2	3	4	5	1	2	3	4	5					
${\hat{\psi }}$	0.18	0.18	0.43	0.18	0.47	0.23	0.21	0.33	0.25	0.54					
se($\hat{\psi }$)	0.02	0.02	0.03	0.02	0.04	0.02	0.02	0.03	0.02	0.04					
Model B$_{4}$															
Day	1					2					3				
Item	1	2	3	4	5	1	2	3	4	5	1	2	3	4	5
${\hat{\psi }}$	0.20	0.29	0.51	0.31	0.60	0.26	0.25	0.32	0.19	0.43	0.17	0.23	0.33	0.18	0.51
se($\hat{\psi }$)	0.02	0.02	0.04	0.03	0.05	0.02	0.02	0.03	0.02	0.03	0.01	0.02	0.03	0.02	0.04
Day	4					5					6				
Item	1	2	3	4	5	1	2	3	4	5	1	2	3	4	5
${\hat{\psi }}$	0.16	0.20	0.27	0.22	0.58	0.18	0.21	0.29	0.17	0.56	0.21	0.20	0.32	0.19	0.49
se($\hat{\psi }$)	0.01	0.02	0.02	0.02	0.04	0.01	0.02	0.02	0.02	0.04	0.02	0.02	0.03	0.02	0.04
Day	7					8									
Item	1	2	3	4	5	1	2	3	4	5					
${\hat{\psi }}$	0.18	0.18	0.43	0.18	0.47	0.23	0.21	0.33	0.25	0.54					
se($\hat{\psi }$)	0.01	0.02	0.03	0.02	0.04	0.02	0.02	0.03	0.02	0.04					
