# Supplementary figures and images for: A Latent Variable Mixed-Effects Location Scale Model with an Application to Daily Diary Data
Source: Psychometrika. 2022 May 3;87(4):1548–70. doi: 10.1007/s11336-022-09864-8 (PMC9636112; doi:10.1007/s11336-022-09864-8)

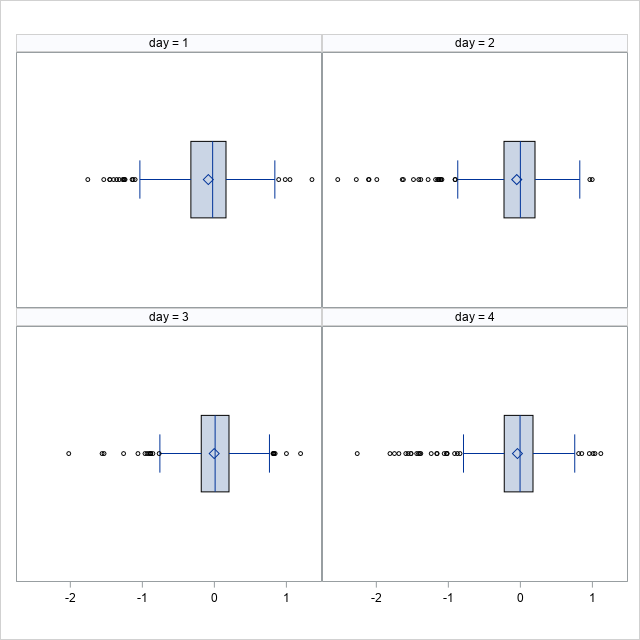


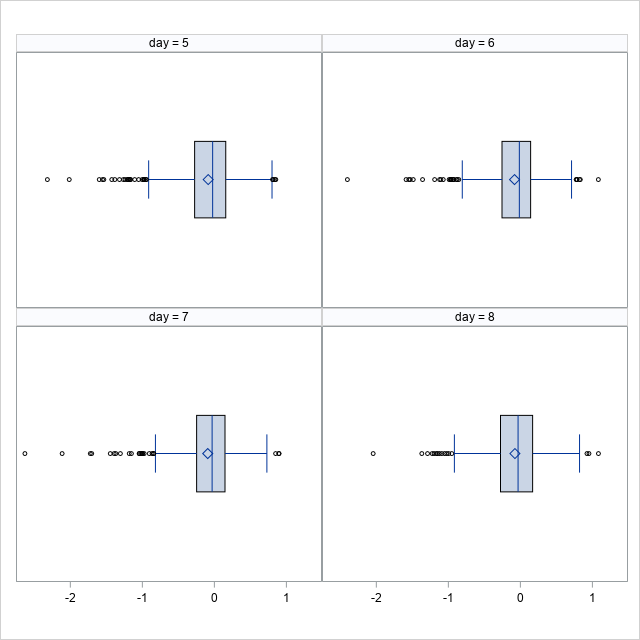


Figure S1. Residuals by interview day based on Model 3b.

Supplement: Supplementary file 1 — (docx 38 KB) [file 11336_2022_9864_MOESM1_ESM.docx]
