# Supplementary material for: A Latent Variable Mixed-Effects Location Scale Model with an Application to Daily Diary Data
Source: Psychometrika. 2022 May 3;87(4):1548–70. doi: 10.1007/s11336-022-09864-8 (PMC9636112; doi:10.1007/s11336-022-09864-8)
Supplement: Supplementary file 2 — (docx 28 KB) [file 11336_2022_9864_MOESM2_ESM.docx]

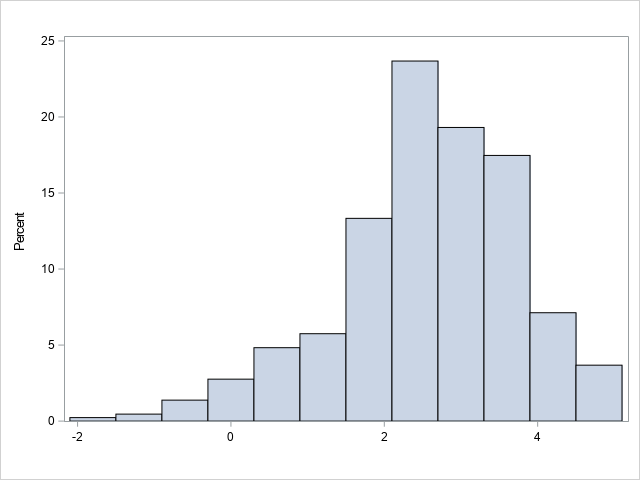


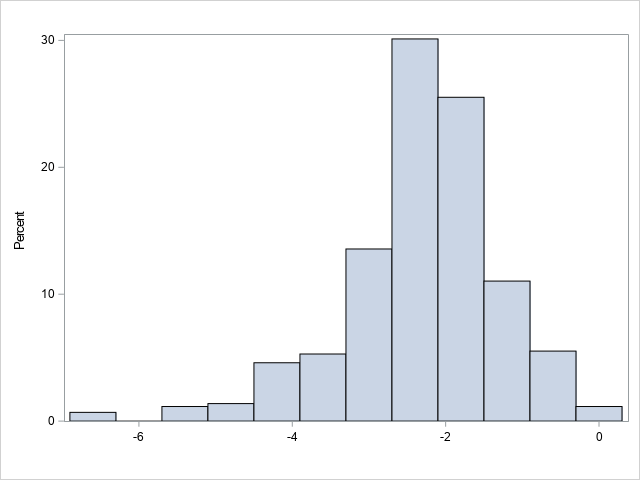


Figure S2. Histograms of the empirical Bayes (EB) estimates of the random intercept (upper figure) and scale effect (lower figure) obtained by a two-step process. Step 1: Factor score estimates (factor score estimates = ${\hat{\boldsymbol{\beta}}}_{0}+\hat{\boldsymbol{\Lambda}}`{\hat{\boldsymbol{\Psi}}}^{-1}\hat{\boldsymbol{\Lambda}}\hat{\boldsymbol{\Lambda}}`{\hat{\boldsymbol{\Psi}}}^{-1}(\boldsymbol{y}_{\boldsymbol{i}}\boldsymbol{-}\hat{\boldsymbol{\tau}}\boldsymbol{-}\hat{\boldsymbol{\Lambda}}\hat{\boldsymbol{\beta}}$)computed from ML estimates of Model B_3._ Step 2: EB estimates of the random intercept and scale effects computed using PROC NLMIXED with Guassian quadrature with 7 quadrature points.
